# Quantification in Experimental Psychology and Pragmatic Epistemology: Tension Between the Scientific Imperative and the Social Imperative

**DOI:** 10.3389/fpsyg.2020.603617

**Published:** 2021-01-13

**Authors:** Hervé Guyon

**Affiliations:** Université de Bretagne Occidentale, AMURE Laboratory, Brest, France

**Keywords:** bio-power, epistemology, measurement, ontology, pragmatism, quantitative psychology, statistical positivism

## Introduction

This article is an opinion article that criticizes the usual practice in quantitative psychology. Our development seeks to link a (pragmatic) critique of measurement and statistical modeling, by considering that the critique must firstly focus on the current social framework of scientific production.

The mainstream of quantification in experimental psychology continues to generally use a standardized design, labeled *statistical positivism* (Gigerenzer, 1990b). Quantification requires quantitative measures. Most articles using such measures do so as if these attributes could be measured like the objects studied in physics. Based on these measures, statistical models are used with different problems: (1) confusion between reality, concepts, and variables; (2) errors in the analysis or interpretation of statistical models; and (3) normative vision of the model that neglects singularities and the interdependence of individuals. Criticism of the positivist claims of empirical studies in psychology has been around for a long time. Why does experimental psychology continue to proceed as if this critique did not exist? The fundamental reason is the social function of quantitative psychology. Statistical models allow researchers to publish so-called scientifically valid results (publication bias). Beyond the scientific field, scientific results in psychology contribute in the public space to what Foucault called *bio-power* (Foucault, 1995): the results of experimental psychology not only serve to support public health recommendations but also underpin processes of standardization, control, and regulation.

Because psychology science is different from what it is in natural science (Hacking, 2000), we have to break away from the dominant social practice in psychology. Considering that “Pragmatism starts from the premise that ‘thinking is for doing’ […] A pragmatist philosophy of science urges scientists to observe what behaviors emerge in the complexity of real life; it encourages active theorizing about individuals’ contexts and the way that individuals construe or interpret them” (Gantman et al., 2018, p. 4), measurement and statistical modeling in psychology should be seen as part of a pragmatic approach and not as a protocol proving theoretical hypotheses on individual psychological dynamics.

## The Nature of the Psychological Attributes and the Measurement Issue

The focus of our critique of measurement in psychology is the object being explored by a measurement. Mainstream psychology considers that measuring a mental attribute amounts to considering that a psychological attribute is a true reality, independent of the knower, that can be located physically, in the same way as physics is able to locate its objects. Psychology must break with the dominant epistemology of “biological realism” (Lloyd, 2010; Zachar, 2010). This does not mean that we must return to an instrumentalist/constructivist epistemology. We consider that, in psychology, we need to adopt a pragmatist and realist epistemology (Maul, 2013; Guyon et al., 2018). In psychology, we seek to measure psychological attributes that are real objects but need to be apprehended through the prism of social practice; their ontological nature is different from the objects that physics measures (Searle, 1996). A psychological attribute is an emergent property (Maul, 2013; Held, 2014; Maul et al., 2016; Guyon et al., 2017), the reality of a psychological attribute resides in its functional manifestation (Maul, 2013). A concept in psychology can thus be considered as referring neither to a fixed reality (external to social praxis) nor to a singular construction independent from physical reality (Maul, 2013; Guyon et al., 2017). The categories used in psychology are relational entities, interactive genres (Hacking, 2000). This necessary theory of knowledge for us relates to pragmatism, not in the common sense of the term, but in the acceptation of the philosophy of science (Guyon et al., 2017, 2018; Maul and McGrane, 2017). Pragmatism-realism does not deny the objectivity of knowledge, even if it is a practical objectivity (Maul, 2013; Guyon et al., 2017, 2018). Apprehending reality as being subjectivated is not in contradiction with the consideration that we objectivate reality (Putnam, 1981, 1992), even if the process of objectification is carried out through the prism of tools of representation (language or other).

In consequence, a protocol for validating measurement specific to the field of psychology is therefore needed, breaking with the formal framework of measurement in physics. Such a protocol to validate a measurement in psychology appears to be operational if it is considered as a pragmatic approach (Sherry, 2011; Mari et al., 2012; Guyon et al., 2018; Maul et al., 2018).

## Statistical Modeling Issue

The term *construct*, since Cronbach and Meehl (1955), has generally been used in psychology to characterize mental attributes in quantitative models. Academic literature points to a confusion on how to apprehend a construct in empirical studies (Slaney, 2001; Borsboom et al., 2009; Lovasz and Slaney, 2013; Maraun and Gabriel, 2013; Markus and Borsboom, 2013; Michell, 2013; Slaney and Racine, 2013). Clear and precise definition of a construct is rare in psychology because of the confusion between concept, variable, and reality (Maraun and Peters, 2005; Maraun and Gabriel, 2013). The statistical model is an abstract and formal representation of associations between variables (mathematical formalism). A clear distinction must be made between the reality (the real psychological attribute), the associated concept (which categories the psychological attribute), and the mathematical formalization of the psychological attribute using a variable. This tension between psychological attribute, concept, and variable generates tensions between substantive theory and statistical model. The statistical model represents the theory in mathematical representation, but there is no equivalence between the two. From the statistical model to the theory, there is the addition of “meaning,” that is to say that we move from a mathematical formalism to the substantive theory (Falissard et al., 2013). In addition, when a statistical model is considered validated, there is no statistical method to consider that it correctly models the operationalized objects because of the potential effects of confounding variables or the problem of equivalent models (models with the same statistical validity but with very different theoretical meanings). It is the scientist, in relation with the substantive theory, who will discuss the reasons for considering one model as relevant. Moreover, any normative model neglects singularities and the interdependence of individuals. The empirical regularities detectable by the statistical methods in psychology never constitute knowledge that can be applied to individuals without discussion (Danziger, 1985; Molenaar, 2004; Borsboom et al., 2009; Borsboom and Markus, 2013; Lamiell, 2013, 2019).

The reason why experimental psychology transforms an average model into a valid model for each individual is expressed as follows: “in the thrall of a physical of science and, as a consequence a physical image of man, psychology was forced to eliminate the particular individual” (Gigerenzer, 1990a, p. 29). Statistics in psychology aim to align with the ideals of *determinism* and *objectivity* (Gigerenzer, 1990a). What drives this statistical positivism in psychology stems from three beliefs about quantitative models: (a) that they are intrinsically objective, ensuring objectivity in research work, (b) that they provide estimated values with precision (through fit indicators), and (c) that they ensure scientific rigor (Tafreshi et al., 2016). Therefore, the scientific ideal of physics has become the general ideal and still serves as a reference.

Various articles return to the construction of the quantitative imperative in psychology and the positivism/modernism underpinnings (Danziger, 1985; Gigerenzer, 1990a; Martin, 2003; Michell, 2003a,b). In fact, the results of a psychological statistical model depend on subjective choices: choice of coding of variables, choice of model, and choice of interpretation of results. A psychological statistical model poses a *prototype* (average model) to which no one corresponds and it serves as a kind of *ideal-type* (Niaz, 2005). Moreover, the meaning of a statistical model underlies our philosophical/theoretical beliefs and commitments (Allen and Clough, 2015). We consider that quantitative models are operational in psychology if they are part of a pragmatic approach.

## A Social Issue

Danziger (1985) and Porter (1996) showed how quantitative methods were introduced into public life because they were thought to embody the qualities of objectivity and trustworthiness, with an implicit belief in the scientific neutrality of the techniques used. Statistical methodology has become highly institutionalized, providing important criteria for publication policies that became methodological imperatives in the academic literature, even if these methods are erroneous or misapplied (Danziger, 1985; Gigerenzer, 1990a; Lambdin, 2012). There is a rhetoric of scientific language in psychology to foster the authority of knowledge in psychology because it creates the illusion of a scientific validity of results identical to that in the natural sciences (John, 1992).

On the basis of these supposedly objective results, psychological science should be used to help social decisions (Ferguson, 2015). There are many sectors that use such results: tests on children, human resource management, etc. These management tools need to rely on certain results, otherwise their recommendations could appear fragile. Yet, we know that some of the statistical results from psychological quantitative models are wrong; Krantz and Wallsten (2019, p. 132) wrote: “We are horrified by much of the statistical practice in psychology and other research.” However, the critical discourse on psychological models is not prominent because these models respond to a social demand. Psychological models are therefore well-embedded in social contexts and issues. The usefulness of psychological models should be understood in relation to the stakeholders’ issues, for example, the personality scales widely used by companies while the scientific foundations of which are clearly open to criticism (Lamiell, 2000, 2013; Cramer et al., 2012; Franić et al., 2013). Lacot et al. (2015) rightly say that these psychological models are “primarily ideological.” They provide a framework for individuals, where singularity is apprehended only in relation to a norm.

Psychological quantitative models, in the name of objectivity and the determinism of its results, serve as a means of recourse to processes of standardization, control, and regulation, called bio-power by Foucault (1995, 1998). We therefore join Hacking in his critique of the political function of psychology. For Hacking (1998, p. 215), bio-power “engendered the specific technologies of statistics,” and bio-power can be extended to “the mind.”

## Call for a Pragmatic Approach

We are witnessing a production of psychological models whose objective foundations are sometimes/often open to criticism (Toomela, 2008; Lamiell, 2013) and which can only be understood by their social and political functions in discourse. To be recognized as a “science,” the goal of positivist/modernist psychology is to find stable empirical regularities (Tafreshi et al., 2016). When empirically verified, these regularities acquire the status of scientific theories to explain and anticipate the behaviors of individuals (Chirkov and Anderson, 2018). It is indeed the social usage of psychological quantitative models that is criticized, both in the academic field and in the public space. The symbolic weight of statistical results makes it possible to attribute so-called objective facts to individuals, thus making it possible to justify social and political dynamics. Statistical models are more often not actually used to help understand the individual, but they are used for assessment and to set up normative relational frameworks (bio-power). But, this social function of statistical models is not intrinsically linked to the processes of quantitative psychology.

We assert that the scientific approach in psychology must break with the modernist claim. If psychology is a means of intervening in social interaction to support personal approaches (psychiatric pathology, educational assistance, etc.), we must criticize the political function of statistics in psychology. The psychologist must clearly differentiate between singularity and the norm (the average results to which the model refers). More fundamentally, a quantitative model validates an average relational structure between variables (i.e., an abstract codification of real objects) and cannot in itself explain the underlying mechanisms that theory hypothesizes between these real objects (psychological attributes). It must be clearly stated that statistical models can only serve as potential benchmarks, teaching psychologists to distance themselves from these formalizations/representations inscribed in a practice and commitments.

We consider that quantitative models are operational in psychology if they are part of a pragmatic approach: pragmatic approach to conceptualizing psychological attributes, pragmatic approach to measuring psychological attributes, and pragmatic approach to analyzing statistical modeling. We reject the “anything goes” argued by Feyerabend (2008) because there is possible room for quantitative studies in psychology between modernism and post-modernism. By calling on pragmatism and realism, psychology can find the resources to assert itself as a science of the human complex using quantitative studies, breaking with normative practice in academic psychology and normative function in the public space.

## Data Availability Statement

The original contributions presented in the study are included in the article/supplementary material, further inquiries can be directed to the corresponding author/s.

## Author Contributions

The author confirms being the sole contributor of this work and has approved it for publication.

## Conflict of Interest

The author declares that the research was conducted in the absence of any commercial or financial relationships that could be construed as a potential conflict of interest.
