# Erratum: Quantification in experimental psychology and pragmatic epistemology: Tension between the scientific imperative and the social imperative

**DOI:** 10.3389/fpsyg.2022.1026974

**Published:** 2022-09-08

**Authors:** Frontiers Production Office

**Affiliations:** Frontiers Media SA, Lausanne, Switzerland

**Keywords:** bio-power, epistemology, measurement, ontology, pragmatism, quantitative psychology, statistical positivism

The name previously presented as the second author is a fictional character, which is contrary to the authorship policies of Frontiers journals.

This name has been removed, and we have placed it in the acknowledgments instead, written as: “The authors would like to acknowledge Camille Noús, a symbol of scientific collaboration.”

The publisher apologizes for this mistake. The original version of this article has been updated.

## Publisher's note

All claims expressed in this article are solely those of the authors and do not necessarily represent those of their affiliated organizations, or those of the publisher, the editors and the reviewers. Any product that may be evaluated in this article, or claim that may be made by its manufacturer, is not guaranteed or endorsed by the publisher.

